# Modelling infectious disease to support human health

**DOI:** 10.1242/dmm.049824

**Published:** 2022-08-29

**Authors:** David M. Tobin

**Affiliations:** 1 Department of Molecular Genetics and Microbiology, Duke University School of Medicine, Durham, NC 27710, USA; 2 Department of Immunology, Duke University School of Medicine, Durham, NC 27710, USA

## Abstract

During the current COVID-19 pandemic, there has been renewed scientific and public focus on understanding the pathogenesis of infectious diseases and investigating vaccines and therapies to combat them. In addition to the tragic toll of severe acute respiratory syndrome coronavirus 2 (SARS-CoV-2), we also recognize increased threats from antibiotic-resistant bacterial strains, the effects of climate change on the prevalence and spread of human pathogens, and the recalcitrance of other infectious diseases – including tuberculosis, malaria, human immunodeficiency virus (HIV) and fungal infections – that continue to cause millions of deaths annually. Large amounts of funding have rightly been redirected toward vaccine development and clinical trials for COVID-19, but we must continue to pursue fundamental and translational research on other pathogens and host immunity. Now more than ever, we need to support the next generation of researchers to develop and utilize models of infectious disease that serve as engines of discovery, innovation and therapy.

## Abstract (precis)

**Summary:** This Editorial considers how knowledge from animal and other models of infectious disease can impact our understanding of human biology and potential therapies, focusing largely on zebrafish. It also highlights ways in which DMM is supporting these areas.

As an Editor at Disease Models & Mechanisms (DMM) and an academic researcher using zebrafish as a model to study tuberculosis, it is especially exciting to read and publish research in zebrafish to obtain, in a whole, live vertebrate, insights into infectious diseases and therapies (Box 1). Indeed, zebrafish provide a remarkable vertebrate model for many questions related to infectious disease. Embryos and larvae are optically transparent, enabling microscopy of both pathogen and host that would be more challenging or cumbersome in other systems (Fig. 1). Knock-in of fluorescent tags at endogenous loci allows direct and detailed *in vivo* visualization of the host immune response (Cronan et al., 2018). Both forward and reverse genetic approaches for understanding infection are straightforward and are buttressed by the high-throughput capabilities of this model, in which a single tank of adult zebrafish can produce hundreds of embryos per week. Furthermore, chemical biology screens and interventions using intact, living animals are uniquely accessible to researchers, as zebrafish larvae and embryos are permeable to diverse small molecules and fit within a single well of 96-well and 384-well plates (Patton et al., 2021).

**Fig. 1.**
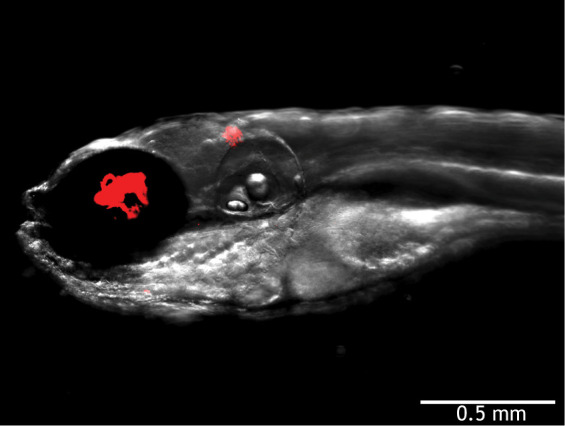
**Zebrafish larva infected with fluorescent *Mycobacterium abscessus* expressing TdTomato, shown in red.** Image courtesy of Matt Johansen (Johansen et al., 2021).

Although efforts in zebrafish are often recognized and valued within the model organism community and beyond, it can sometimes be hard to break through to the world of clinical research. I vividly remember the excitement of being invited to present my work as a starting assistant professor at an early-career researcher lunch with a prominent visiting scientist, only to have my research and plans dismissed with some variation of “Well, why don't you try to figure out what's actually going on in people?”.

Indeed, this is what many zebrafish researchers are ultimately trying to do by a different route. The goal of harnessing the knowledge we generate in models to impact human biology and therapies is an important part of the scientific enterprise. Many of us want and expect our findings to be relevant beyond the context of a model system. In my field, it has been exciting to see work in the zebrafish emerge that has led to the discovery of fundamentally conserved features of tuberculosis and host immunity – from zebrafish to humans – and has since translated to ongoing clinical trials.

However, although we might hope that our work will be inherently understood and utilized in the clinical context, maximizing the potential of this research requires community advocates and communicators to help place the work in context. This can be achieved through ongoing dialogue among researchers, clinicians and patients to understand medical needs and perspectives. For example, DMM and The Company of Biologists have been long-time supporters of societies, such as the Zebrafish Disease Models Society, which focuses on the translational potential of zebrafish for understanding human disease and for developing new therapies, including some being investigated in clinical trials.

Thus, it is useful to consider the following three broad themes when using model organisms in infectious disease research:

1. **Conserved host–pathogen interactions in model systems.** Although we all recognize, even at a strictly visual level, the many differences between the biology of a model organism and human biology, there is fundamentally conserved biology to be explored. Immune signalling pathways and underlying principles, as well as molecular and cellular details, first discovered and dissected in worms, flies, fish, mice and other model organisms, have translated remarkably well to human biology in many cases.
2. **Model diversity.** Divergent biology – in addition to being fascinating and important for the sake of knowledge itself – also leads to vital new insights and therapeutic approaches. As just one example, bacteriophages were instrumental in the discovery of fundamental aspects of gene regulation, have been used to facilitate genetic manipulation of seemingly genetically intractable pathogens, and are now being engineered and deployed therapeutically. And the study of bacterial–bacteriophage interactions of course led to all the advances made possible by CRISPR. These and many other examples from models that diverge from humans all support open-mindedness in science and emphasize the strength of laboratories taking diverse approaches and using diverse models. Pressing questions and opportunities in this realm are many, including investigation of how some non-human immune systems – those of bats, as just one example – permit asymptomatic tolerance of viruses that may be pathogenic in humans (Hayman, 2019). Which animal species restrict human pathogens via immune mechanisms that might eventually be harnessed therapeutically? Some of these topics will be prominent in a 2023 meeting organized by DMM entitled ‘Infectious Diseases Through an Evolutionary Lens’, which will take place in London at the British Medical Association House (Fig. 2).

**Fig. 2.**
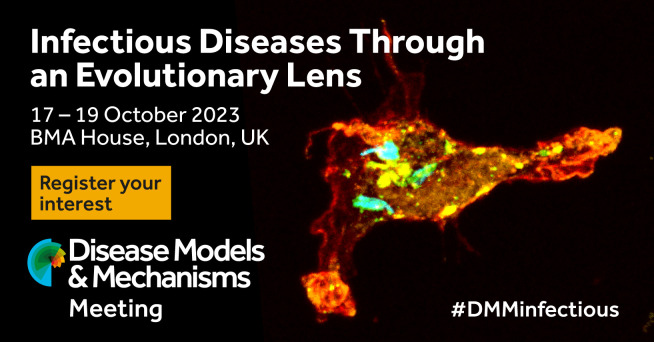
**DMM's 2023 meeting is entitled ‘Infectious Diseases Through an Evolutionary Lens’ and will take place in London at the British Medical Association House.** Register your interest here: https://www.biologists.com/infectious-diseases-through-an-evolutionary-lens-contact-form/.
3. **Engineering preclinical and predictive models of infectious disease.** With advances in gene editing and the ability to make specific base edits, it is possible to precisely model human variants in an *in vivo* context during infection. Organisms like the zebrafish can provide useful models to delve into the specific consequences of these variants. Orthogonal approaches include mammalian animal models and advanced human cell models (Leist et al., 2020; van der Vaart et al., 2021). Discussion between scientists doing this preclinical work and clinical collaborators will be needed to determine to what degree the model recapitulates human disease and how these models can be used to advance new therapies. Recently, we have seen some of the landscape for clinical trials change, and in public health emergencies, collaborations would ideally accelerate the time from discovery to clinic. Again, this will require dialogue with and buy-in from clinical researchers to put together rigorous clinical trials.

DMM seeks to create and contribute to the ongoing conversations among and between basic scientists, clinical researchers and clinicians, with insights and criticisms from each of these domains. By highlighting rigorous, high-quality science in these areas, we hope to contribute to improved understanding of infectious diseases and new approaches to treatment.

**Box 1. DMM highlights zebrafish advancing knowledge in infectious disease**

Recent publications in DMM show the potential of the zebrafish model system to provide new or fuller insights into infectious diseases and therapies. One question being addressed is how basic cell-autonomous immune processes function in the context of a full organism. Various Reviews have highlighted what we have learned about the role of pyroptosis in host defence against bacterial infections (Brokatzky and Mostowy, 2022), as well as advances in understanding the diverse roles that macrophages and neutrophils play during the initial response to a variety of infectious and inflammatory stimuli (Rosowski, 2020). Zebrafish can also provide models for parasitic diseases that are relatively neglected, and we were pleased to publish a zebrafish model that provides insight into *Toxoplasma* pathogenesis, particularly the *in vivo* interactions of *Toxoplasma* with macrophages (Yoshida et al., 2020). Research dissecting the role of host immune cells in pathogen responses can be further potentiated by new tools, such as those developed by the Lieschke laboratory using macrophage and neutrophil-specific Cas9 driver lines to allow cell-specific genetic perturbation (Isiaku et al., 2021).

Non-tuberculous mycobacteria causing pulmonary disease are a growing threat worldwide, with an antibiotic resistance profile that makes them very difficult to treat (Stout et al., 2016; Vinnard et al., 2016). An exploration of phage therapy for non-tuberculous mycobacteria in the zebrafish provides new insights into exciting clinical work that bookends this publication (Johansen et al., 2021). Engineered bacteriophages targeting specific strains of *Mycobacterium abscessus* have now been used clinically in cases of advanced lung disease (Dedrick et al., 2019; Nick et al., 2022). In other work, Habjan et al. employed the zebrafish mycobacterial infection model as an early screening step for anti-tuberculosis hits from *in vitro* screens that might have the best chance for *in vivo* translation. Following up on a screen for novel *in vitro* activity against *Mycobacterium tuberculosis* that identified ∼240 compounds, they identified 14 compounds with good *in vivo* activity. Impressively, they went on to identify the target of the strongest *in vivo* hit as being a mycobacterial aspartyl-tRNA synthase through screening for resistant mutants in both *Mycobacterium marinum* and *Mycobacterium tuberculosis* (Habjan et al., 2021).

Drug screens, like those discussed above, are possible due to the permeability of the zebrafish to small molecules, which also allows creative ways to control the induction of host cytokines. DMM published an approach that enables drug-inducible, tissue-specific, titratable expression of different cytokines (Ibrahim et al., 2020). Harnessing this permeability in zebrafish can also enable detailed exploration of the effects of drugs, such as broadly used glucocorticoids, on specific innate immune cell types (Xie et al., 2019).

The zebrafish has also been used as a model to understand infectious disease therapies targeting the pathogen directly. A recent paper describes the *in vivo* efficacy of nanoparticle-based delivery of lipophilic antibiotics, as well as use of the zebrafish to screen different formulations (Knudsen Dal et al., 2022). Finally, in the adult zebrafish sphere, a recent Review focused on how zebrafish can inform vaccine development strategies (Saralahti et al., 2020).

These recent publications highlight some of the strengths of the zebrafish model for infectious disease research. DMM aims to be at the forefront in encouraging scientists and clinicians to leverage these insights for future therapies.
